# Therapeutic Potential of Vital Transcription Factors in Alzheimer’s and Parkinson’s Disease With Particular Emphasis on Transcription Factor EB Mediated Autophagy

**DOI:** 10.3389/fnins.2021.777347

**Published:** 2021-12-14

**Authors:** Sachchida Nand Rai, Neeraj Tiwari, Payal Singh, Divya Mishra, Anurag Kumar Singh, Etrat Hooshmandi, Emanuel Vamanu, Mohan P. Singh

**Affiliations:** ^1^Centre of Biotechnology, University of Allahabad, Prayagraj, India; ^2^Faculty of Biosciences, Institute of Biosciences and Technology, Shri Ramswaroop Memorial University, Barabanki, India; ^3^Department of Zoology, Mahila Mahavidyalaya, Banaras Hindu University, Varanasi, India; ^4^Centre of Bioinformatics, University of Allahabad, Prayagraj, India; ^5^Centre of Experimental Medicine and Surgery, Institute of Medical Sciences, Banaras Hindu University, Varanasi, India; ^6^Clinical Neurology Research Center, Shiraz University of Medical Sciences, Shiraz, Iran; ^7^Faculty of Biotechnology, University of Agronomic Science and Veterinary Medicine, Bucharest, Romania

**Keywords:** Parkinson’s disease, Alzheimer’s disease, TFEB, autophagy, NF-κB

## Abstract

Autophagy is an important cellular self-digestion and recycling pathway that helps in maintaining cellular homeostasis. Dysregulation at various steps of the autophagic and endolysosomal pathway has been reported in several neurodegenerative disorders such as Alzheimer’s disease (AD), Parkinson’s disease (PD), and Huntington disease (HD) and is cited as a critically important feature for central nervous system (CNS) proteostasis. Recently, another molecular target, namely transcription factor EB (TFEB) has been explored globally to treat neurodegenerative disorders. This TFEB, is a key regulator of autophagy and lysosomal biogenesis pathway. Multiple research studies suggested therapeutic potential by targeting TFEB to treat human diseases involving autophagy-lysosomal dysfunction, especially neurodegenerative disorders. A common observation involving all neurodegenerative disorders is their poor efficacy in clearing and recycle toxic aggregated proteins and damaged cellular organelles due to impairment in the autophagy pathway. This dysfunction in autophagy characterized by the accumulation of toxic protein aggregates leads to a progressive loss in structural integrity/functionality of neurons and may even result in neuronal death. In recent years TFEB, a key regulator of autophagy and lysosomal biogenesis, has received considerable attention. It has emerged as a potential therapeutic target in numerous neurodegenerative disorders like AD and PD. In various neurobiology studies involving animal models, TFEB has been found to ameliorate neurotoxicity and rescue neurodegeneration. Since TFEB is a master transcriptional regulator of autophagy and lysosomal biogenesis pathway and plays a crucial role in defining autophagy activation. Studies have been done to understand the mechanisms for TFEB dysfunction, which may yield insights into how TFEB might be targeted and used for the therapeutic strategy to develop a treatment process with extensive application to neurodegenerative disorders. In this review, we explore the role of different transcription factor-based targeted therapy by some natural compounds for AD and PD with special emphasis on TFEB.

## Introduction

### Factors Modulating Alzheimer’s Disease

Neurodegenerative diseases are a multifactorial heterogeneous group of nervous system disorders characterized by structural/functional dysfunction of neurons in the brain (Sheikh et al., 2013). The resulting selective and progressive, irreversible neuronal loss and degeneration leads to incurable neuro-pathological conditions (Hussain et al., 2018). Neurodegenerative disorders may involve selective vulnerability and most often are associated with diverse clinical manifestations varying from memory impairments and cognitive decline, eventually affecting behavior, speech, and the motor system (Dugger and Dickson, 2017). One of such most common neurodegenerative disorders is Alzheimer’s disease (AD). AD is the most common form of dementia globally, with an irreversible and progressive cerebral decline involving behavioral and cognitive functions including memory, language, and judgment, ultimately resulting in total dependence on others, thus becoming a major global public health concern (DeTure and Dickson, 2019).

In earlier stages of AD, there is a gradual and progressive decline in cognitive functions that involves memory and other cognitive domains, such as visuospatial functions and language (Tarawneh and Holtzman, 2012). Genetically, AD has been classified in familial and sporadic forms (Klein and Westenberger, 2012). The apolipoprotein E gene (APOE) has been identified as a major genetic risk factor causing sporadic cases of AD, while amyloid precursor protein (APP), presenilin 1/2 (PSEN1/2) genes, along with other co-morbidity factors, such as Triggering receptor expressed on myeloid cells 2 (TREM2), ATP- binding cassette transporter 1 (ABCA1), ATP-binding cassette transporter7 (ABCA7), Methylenetetrahydrofolate Dehydrogenase 1 (MTHFD1), Bridging Integrator 1 (BIN1) and gamma-secretase, are associated with Familial AD (Calero et al., 2015; Lanoiselée et al., 2017). Currently, there is no effective cure, although few licensed treatments are available that can help attenuate AD symptoms. At present, nearly 35.6 million patients are believed to be affected by AD worldwide, and about 4.6 million new cases have been added annually, causing an enormous socioeconomic burden. Therefore, there is a pressing need to understand the mechanisms of AD pathogenesis and fast-track the discovery of novel therapeutic targets (Yiannopoulou and Papageorgiou, 2013). It is a well-known fact that AD, like most other neurodegenerative diseases, is fundamentally a proteinopathy, characterized by two pathologies, i.e., altered protein folding and aggregation of two proteins, namely amyloid β-protein (Aβ) and tau (Holtzman et al., 2012; Ashraf et al., 2014).

Amyloid plaques are insoluble aggregates of the β-amyloid protein (Aβ). Aβ peptides are formed by proteolytic cleavage of APP resulting in the formation of Aβ_1–40_ and Aβ_1–42_ (Murphy and LeVine, 2010). Initially, Aβ accumulation starts from the temporal lobe region of the brain and later gets diffused to the entire cortex region during the final stages of the disease. This deposition and accumulation of amyloid plaques result in synaptic dysfunctions, inflammatory responses, and neuronal dysfunction that ultimately causes neurodegeneration, a major characteristic of AD and correlates with clinical symptoms (DeTure and Dickson, 2019). Tauopathies represent another major hallmark of AD. In AD this tau pathology is represented by neurofibrillary tangles (NFTs), which are aggregations of abnormally hyper-phosphorylated tau protein and can disrupt neuronal structure and function (Iqbal et al., 2010).

In addition to amyloid plaque and tau proteins, cholinergic neurons located in the basal forebrain are severely lost and degenerated due to alterations in presynaptic cholinergic terminals in the hippocampus and neocortex in AD, which are important for memory disturbances and other cognitive symptoms (Ferreira-Vieira et al., 2016). This cholinergic disruption has been proven by the use of acetylcholine esterase inhibitors (AChEI), which increase the level and duration of neurotransmitter action by inhibiting the acetylcholinesterase enzyme from degrading acetylcholine in the synaptic cleft (Colovic et al., 2013).

Apart from this, there is still a lacuna in detecting and sensing different protein conformers (monomers, oligomers, and fibrils) of Aβ, tau, and other proteins that are known to aggregate in neurodegenerative diseases further create hurdles. Therefore there is an urgent need to understand the pattern and development stages of different biochemical, cellular, and neurovascular abnormalities over time that contribute to brain dysfunction, synaptic dysfunction, inflammation, cognitive impairment, and neuronal death in AD. Although the presence of amyloid plaque deposition and NFTs have been employed as a rationale for possible treatments for AD, the involvement of other factors involved in the pathogenesis and influencing disease development can’t be ruled out. Beyond Aβ and tau, how oxidative stress, brain injury/ischemia, genetic and age-related factors contribute to the progression of neurodegeneration needs to be properly understood at the individual and cellular levels (Mattson and Arumugam, 2018). Also, there is a need to identify and target potential receptors and signaling pathways that may help in our understanding of how Aβ accumulation leads to altered and impaired function and neuronal processes.

Several studies have discovered these alternate factors and pathways; however, the underlying mechanisms are still not completely understood. Importantly, we also need to understand the brain circuitry and synaptic transmission at both micro and macro levels during brain dysfunction at different stages of AD pathology.

## Current Treatment Options for Alzheimer’s Disease

**FIGURE 1:**
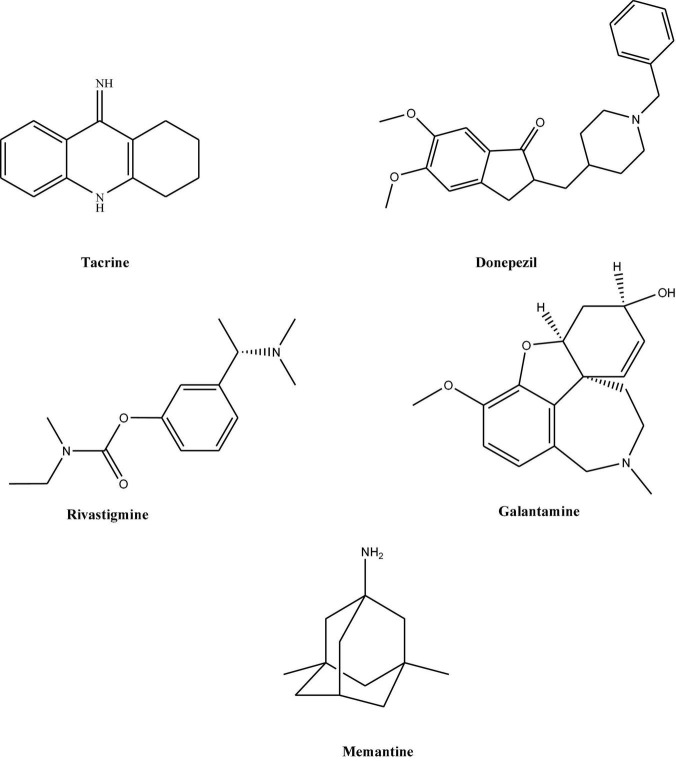
Chemical structures of various therapeutic compounds for the treatment of AD.

As explained earlier, AD is a progressive neurodegenerative disease, for which currently no permanent therapeutic option is available. However, concerning AD, there are very few drugs available for the treatment of symptomatic cases. These medications include mainly cholinesterase inhibitors (Grutzendler and Morris, 2001). Since 1990 five drugs namely; tacrine, donepezil, rivastigmine, and galantamine, have been available (Hansen et al., 2008; Figure 1). Since Tacrine was associated with significant liver toxicity, therefore its prescription is stopped. All these four medications are classified together as acetylcholinesterase inhibitors and officially approved for treating mild to moderate stages in AD. The last drug, memantine (Figure 1) belongs to N-methyl-D-aspartate (NMDA) receptor antagonists and works by blocking the NMDA receptors in the brain that glutamate binds (Olivares et al., 2012). In addition to this, still, there is no firm criterion suggesting how to best treat an individual with the drugs mentioned above and how these drugs will work best in a particular patient. However, these have been divided as per advancement into AD stages. Most commonly, memantine is given in the treatment of mild to moderate stage.

Also, few studies suggest the use of combination therapy of memantine with donepezil or other cholinesterase inhibitors for moderate to severe AD. For severe or late-stage disease, Donepezil has been approved (Riverol et al., 2011). Donepezil is also beneficial at smaller doses, whereas rivastigmine is effective only at a medium or higher dose (Rösler et al., 1999). However, irrespective of their neurotransmitter effects, these choline mentioned above esterase inhibitors and NMDA antagonists cannot reverse the underlying brain degeneration in AD. Therefore, they are best-considered palliative rather than curative treatment.

In addition to this, adverse effects like nausea, vomiting, dizziness or diarrhea are most likely to happen with the medications mentioned above. The higher the dose, the more likely chances are to occur. Also, all these drugs have limited effectiveness in patients. Cost-effectiveness is also another major hurdle faced by millions of sufferers. The currently available treatment options are symptomatic, i.e., they help improve cognitive and behavioral symptoms without influencing the course of Alzheimer’s disease. So far, research has shown that these medications can temporarily slow down some symptoms for the time being or delay their onset during mild to moderate stages of Alzheimer’s.

Currently, there is no effective treatment for AD that can reverse or slow down its progression as most of the discovered drugs are not designed to treat the underlying cause of AD. During the last few decades, remarkable advances in the field of AD have made it possible to unravel the underlying biological and molecular mechanisms of AD. Despite this, only slight progress has been made, we are still far from developing truly disease-modifying treatment. What are the Possibilities with current ongoing researches, while the answers may not be clear; we believe these researches may pave the foundation for treating all neurodegenerative disorders. Therefore, there is a consistent need to search for potential risk factors contributing to Alzheimer’s pathogenesis.

## Vital Transcription Factors in Alzheimer’s Disease

Transcription factors are sequence-specific DNA-binding protein molecules that regulate gene transcription by binding to the promoter region of genes (Mitsis et al., 2020). The majorities of Transcription factors have at least one DNA-binding domain (DBD). They can either work alone or coordinated in a complex by promoting or blocking the RNA polymerase (Boija et al., 2018).

In AD, there is progressive memory loss and impairment of cognitive function, which ultimately leads to neuron death. The causes of neuron death remain unknown, although research studies of protein conformation, neurotransmitters and their receptors suggest that aberrant protein toxicity and signal transduction leads to cellular dysfunction, producing neuronal cell death (Rajmohan and Reddy, 2017). In addition, oxidative stress and mitochondrial dysfunction are also known to accompany AD pathogenesis (Picca et al., 2020). Nevertheless, various antioxidant therapies against reactive free radicals have been developed and used; their results are ineffective. Since Reactive oxygen species (ROS) also act as signaling molecules and help in redox regulation of various transcription factors, an alternative approach is the need of current time (Uttara et al., 2009). Thus, chronic exposure to ROS in AD could trigger signaling cascades that are regulated by various transcription factors.

In recent years researchers have identified several transcription factors such as activator protein-1 (AP-1), nuclear factor κB (NF-κB), SP 1, and Nuclear factor-erythroid factor 2 (Nrf 2) (Shi and Gibson, 2007). Transcription factor EB (TFEB) is also an important agent that plays a vital role in redox-dependent and autophagy regulation and is activated by several different stimuli such as cytokines, lipopolysaccharide (LPS), and oxidative stress during inflammatory events in neurodegeneration (Brady et al., 2018). Since these transcription factors and mutations inside them have been discovered to be the root cause of many diseases and also their involvement has been found in many neurological disorders, they have become potential therapeutic targets for the development of effective drugs and medicines.

Several studies prove the role of these transcription factors in various mechanistic pathways such as neuroinflammation, oxidative stress, protein misfolding. In this review, we summarize the role of few transcription factors and their possible role in pathological features in Alzheimer’s disease and later with the most important and currently most studied transcription factor TFEB and its role in amyloid-beta and tau pathology.

### Nuclear Factor-κB

The transcription factor nuclear factor-κB (NF-κB) is a redox-sensitive transcription factor playing a diversified role in pro-inflammatory and protective immunity pathways and is known to regulate the expression of nearly 150 genes, including transcription of cytokine antioxidant gene (Panday et al., 2016; Lingappan, 2018). In neurons, NFκB consists of p50, p65/RelA, and I-κB subunits. Normally, NFκB is localized in the cytoplasm in an inactive form bound with the inhibitory proteins known as inhibitors of κB (I-κB) (Giridharan and Srinivasan, 2018). Upon receiving stimulus such as oxidative stress, I- κB gets phosphorylated. This leads to the translocation of NFκB into the nucleus where it binds to a consensus sequence in the promoter regions of target genes (Oeckinghaus and Ghosh, 2009). Furthermore, age-related oxidative stress has been found to regulate the dissociation of I-κB and nuclear translocation of NFκB (Kaltschmidt et al., 1999; Lingappan, 2018). Recent studies show that aging and tissue-specific brain inflammation mediated by NF-κB is associated with each other (Kounatidis et al., 2017). However, NF-κB is highly induced by ROS, ionizing radiation, cytokines such as interleukin 1-beta (IL-1β) and tumor necrosis factor-alpha (TNF-α), and lipopolysaccharides (LPS) (Yin et al., 2002; Kany et al., 2019). In addition to this, NF-κB in the nervous system is stimulated by growth factors and glutamate (Dresselhaus and Meffert, 2019).

In a study involving rats, expression of NF-κB along with neurodegenerative pro-inflammatory enzymes COX-2 cyclooxygenase−2 (COX-2) and inducible nitric oxide synthase (iNOS) was found to be upregulated with aging (Kim et al., 2010). These changes were validated using the anti-inflammatory *Lactobacillus pentosus var. plantarum* C29 in the treatment of aged rats. C29 suppressed the expression of COX-2 and iNOS and the activation of NF−κB in the hippocampus and ameliorated aging-dependent memory impairment by inhibiting the NF−κB signaling pathway (Jeong et al., 2015). In another study involving mice, a positive link was discovered between NF-κB expression and hippocampal neuronal apoptosis (Niu et al., 2010). In addition to this increased expression of NFκB has also been discovered in neurons and astrocytes associated with Aβ plaques in the brains of AD patients (Capiralla et al., 2012).

### AP-1

AP-1, a heterodimeric transcription factor composed of the Jun, Fos, and ATF gene families regulates gene expression in response to several stimuli such as cytokines, UV irradiation, growth factors, oxidative stress, and depletion of intracellular glutathione and is known to be regulated by intracellular redox state (Kim et al., 2013; Garces de los Fayos Alonso et al., 2018). Once activated, AP-1 induces phosphorylation of the JNK pathway leading to transcription of genes involved in both proinflammatory and antioxidant pathways (Shi and Gibson, 2007). It has been found that AP-1 and NFκB together induce the expression of several target genes such as heme oxygenase-1 (HO-1), interleukin-8 (IL-8), and intercellular adhesion molecule (ICAM) that are involved in neuroinflammatory and antioxidant defense pathways associated with AD pathology (Oguro et al., 1996; Lakshminarayanan et al., 1998).

In a study involving Smac mice, Amyloid βeta (25–35) treatment caused increased activation of AP-1 and subsequent expression of Bim, a proapoptotic protein. Later, when Bim was knocked out it suppressed amyloid beta-induced Smac release and resulted in attenuation of cerebral endothelial cells death, suggesting AP-1induction by Aβ, which is one of the major reasons behind AD (Yin et al., 2002).

### Specificity Protein-1

Sp1 is a redox-sensitive transcription factor belonging to the Sp/KLF family, a zinc-finger family of DNA-binding proteins. It consists of 785 amino acids with a molecular weight of 81kDa and binds to GC- rich motifs through its C2H2 zinc finger motif region (Vellingiri et al., 2020).Sp1, together with other pro-inflammatory transcription factors such as c-Jun/AP-1, Nrf2, NF-κB, and p53, have been found to regulate the expression of prominent genes in AD such as amyloid-beta precursor protein (APP) and beta-secretase 1 (BACE1) (Christensen et al., 2004; Docagne et al., 2004; Citron et al., 2015). It is reported that Sp1 plays a significant role in regulating several AD-related proteins, including APP and tau. It could also be regulated by IL-1β cytokine which is an important factor associated with AD pathogenesis. In a study done by Citron et al. (2015), an enhanced expression of Sp 1 mRNA was observed in AD’s frontal cortex, indicating the involvement of transcription factor Sp1 in AD.

### Peroxisome Proliferator-Activated Receptor γ

Peroxisome proliferator-activated receptor γ (PPARγ) is a transcription factor belonging to the nuclear receptor family of Peroxisome proliferator-activated receptors (PPARs) (Zieleniak et al., 2008). The accumulation of Aβ can leads to synaptic dysfunction, initiation of NFTs, neuroinflammation, axonal injury, and synaptic loss (Rajmohan and Reddy, 2017). It is reported that PPARγ regulates the expression of genes involved in adipogenesis, lipid metabolism, inflammation, and maintenance of metabolic homeostasis (Corrales et al., 2018). PPARγ has been found to have a suppressive role on BACE1 expression via the PPARγ-responsive element present in the promoter region, which inhibits Aβ production (Wang et al., 2013; Khan et al., 2019). In a study, PPARγ activation was able to suppress inflammatory cytokine expression, iNOS expression, and NO production in microglial cells and also inhibited COX2 suggesting antagonism toward transcription factor NFκB activity (Combs et al., 2000; Heneka et al., 2011). In another study involving a rat model of cortical Aβ injection, administration of PPARγ agonists such as ciglitazone, ibuprofen, and pioglitazone potently suppressed Aβ-evoked microglial cytokine generation, thus suggesting PPARγ as a potential therapeutic target (Heneka et al., 2003).

### Nrf2

Nrf2 is a cytoplasmic protein bound to sulfhydryl-rich cytosolic protein, Kelch ECH associating protein (Keap1) (Reddy, 2013; Reddy et al., 2016). Keap1, a Cul3-based E3 ligase, targets Nrf2 for subsequent proteasomal degradation to maintain lower intracellular levels (Lipinski et al., 2010). Oxidative or electrophilic stress reacts with Keap1 and represses its activity thus stabilizing Nrf2, leading to increased nuclear accumulation of Nrf2 (Itoh et al., 2001). Once translocated to the nucleus, Nrf2 forms a heterodimer with small Maf proteins and binds to the ARE, to activate transcription of several detoxifications and antioxidant genes (Tonelli et al., 2018).

In several neurodegenerative disorders, modulation of NF-E2 related factor 2 (Nrf2) activities has been observed significantly. Hence, it has become a potential therapeutic target for various neurological diseases such as Alzheimer’s disease, Parkinson’s disease, and Amyotrophic lateral sclerosis (Joshi and Johnson, 2012). Disruption of Keap1-Nrf2 interaction or alterations of Nrf2 activity can increase the endogenous antioxidant/detoxifying systems of the brain, thus maintaining a redox balance and providing protection against oxidative stress in various neurodegenerative disorders (Tu et al., 2019).

### Transcription Factor EB

The TFEB belongs to the microphthalmia/transcription factor E (MiT/TFE) family of helix–loop–helix-leucine-zipper (bHLH- Zip) proteins. TFEB regulates the expression of genes involved in the autophagy–lysosomal pathway (Puertollano et al., 2018; La Spina et al., 2021). It also regulates autophagic flux by promoting and regulating lysosomal and autophagosome biogenesis and regulating autophagosome-lysosome fusion, thereby; facilitating cellular clearance (Settembre et al., 2011). TFEB has also been found to induce selective autophagy and lysosomal exocytosis. In mice models of Parkinson’s and AD, overexpression of TFEB resulted in enhanced degradation of bulk autophagy substrates and help in the clearance of damaged mitochondria and lipid droplets. This overexpression of TFEB also resulted in the expression of several phenotypes known to promote autophagy and lysosomal biogenesis in various diseases (Nezich et al., 2015). Therefore the regulation of autophagy and lysosomal biogenesis pathways by TFEB make it a potential target for the development of neuro disorders therapeutics.

Autophagy is an evolutionarily conserved cellular self-digestion pathway that is constitutively active in CNS (Komatsu et al., 2006; Mizushima et al., 2008). Loss of autophagy in the central nervous system (CNS) causes neurodegeneration in mice. It plays an important role by eliminating toxic proteins and damaged organelles, thereby preventing the accumulation of aggregates and supporting neuronal plasticity. Autophagy has emerged as especially important for CNS proteostasis as its dysregulation has been documented to be associated with neurodegenerative disorders like AD, Parkinson’s disease (PD), and Huntington’s disease (HD) (Cortes and La Spada, 2019). Recently, another molecular target for neurodegenerative disorders has been identified. This discovery of molecular target TFEB as a master regulator of the autophagy-lysosomal pathway has emerged as a potential therapeutic target. Currently, several research studies are focused on exploring its role in treating neurodegenerative disorders involving autophagy-lysosomal dysfunction (Settembre et al., 2013b). Recently, increasing research studies have been designed and performed to understand the function of TFEB in cellular processes. However, studies aiming at identifying the role of TFEB for its potential involvement in neuronal homeostasis are still in the naïve phase (Settembre et al., 2013a; Binder et al., 2020). TFEB is highly expressed in the CNS and is active in both neurons and astrocytes (Bao et al., 2016).

Thus, few studies have been focusing on correlating TFEB function with neurodegeneration. In few studies, TFEB dysfunction and localization have been reported in animal models of AD. Similarly, an analysis of AD patient lymphocytes and monocytes revealed decreased TFEB expression, suggesting its possible role in lysosomal deficits observed in AD patients (Tiribuzi et al., 2014). In agreement with this, the study also observed loss of nuclear TFEB in AD patient brain samples (Wang et al., 2016b). A Similar nuclear exclusion of TFEB was also discovered in an *in vitro* model of double presenilin knock-out cells (Reddy et al., 2016). The effect of TFEB dysfunction on AD pathogenesis as reported by the above studies by cell-specific knockouts and overexpression of TFEB in the CNS may reveal important information in delineating the role of TFEB dysfunction in AD pathogenesis (Cortes and La Spada, 2019).

**FIGURE 2:**
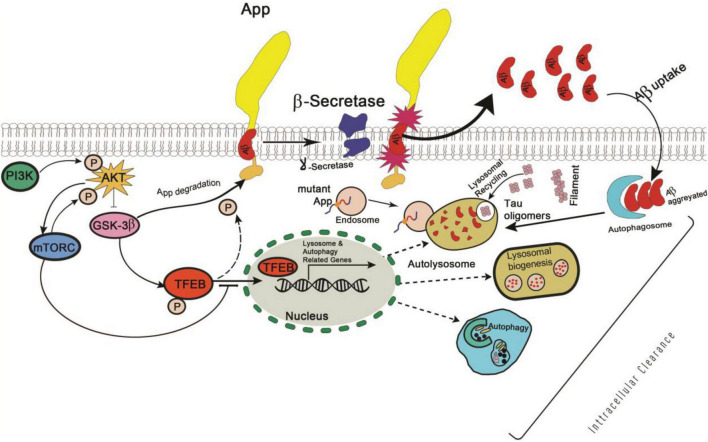
Schematic representation of PI3K/AKT/GSK-3β mediated phosphorylation signaling pathway in Alzheimer’s disease.

Similarly, Glycogen synthase kinase 3 (GSK3), a serine-threonine kinase, was identified as a putative regulator of TFEB activity in 2011 and was found to play a role in AD pathogenesis by phosphorylating APP and tau to promote Aβ42 production (Settembre et al., 2011; Reddy, 2013). Therefore, further studies are required to determine whether TFEB is a direct target of GSK-mediated phosphorylation (Figure 2), and, if this pathway is relevant to *in vivo* AD pathogenesis.

A study involving the alteration of Beclin-1 gene dosage and mTOR inhibition revealed the dysregulation of autophagy transcription networks in AD (Lipinski et al., 2010). Therefore, if neuroprotective induction of autophagy in AD brains needs to work properly, it may require initiation and boost of the entire autophagy pathway, i.e., from the vesicle formation to lysosomal fusion and degradation. Since currently, available autophagy treatments target only autophagy activation, they cannot increase overall lysosomal clearance. In this regard, TFEB can act as an ideal therapeutic target, as it up-regulates autophagy induction and promotes increased lysosomal numbers, function, and clearance (Sardiello, 2016). In agreement with this, TFEB has shown promising results to AD models and effectively reduced neurofibrillary tangle pathology and rescued behavioral phenotypes, synaptic deficits, and neurodegeneration in the mouse model without causing any harmful effects on neurons (Polito et al., 2014).

In a study involving a P301S tauopathy mouse model, neuron-targeted TFEB overexpression in the significantly reduced toxic p-Tau and lipofuscin levels in the cortex and hippocampus region with significantly improved cognitive features (Wang et al., 2016a). Thus, targeting TFEB activity along with autophagy pathways could be a novel therapeutic strategy for AD patients. However, currently available TFEB activators act as MTOR (mechanistic target of rapamycin (serine/threonine kinase) inhibitors, a master regulator of cellular growth and metabolism. Thus, there is a need to identify TFEB modulators that can act without inhibiting the mTOR pathway and probably less deleterious to the cells.

## Effect of Transcription Factor EB on Aβ Pathology

All neurodegenerative diseases, including AD; are mainly manifested by the hallmark feature of amyloid-b (Aβ) deposition leading to a chronic neuronal degeneration in the brain (Murphy and LeVine, 2010). Amyloid-βeta peptide consists of nearly ∼40–42 amino acid residues in length (i.e., Aβ40 and Aβ42). Both Aβ 40 and Aβ 42 are cleaved from much larger APP by b-site APP-cleaving enzyme 1 and the γ-secretase complex (Chow et al., 2010). Among these two forms; Aβ42 is considered highly toxic in neuronal diseases as it can easily self-aggregate into insoluble fibrils compared to Aβ40 (Chen et al., 2017).

In AD, Aβ deposition is mainly linked with defective autophagy (Liu and Li, 2019). Increasing evidence suggest autophagy dysfunction in the pathogenesis of numerous neurodegenerative diseases. Autophagy is an evolutionary conserved, dynamic cellular self-digestion pathway that involves initiation, sequestration, trafficking, lysosomal fusion, and finally lysosomal degradation, also known as autophagic flux (Singh et al., 2018). In the autophagy pathway, lysosomes are critical cellular digestive compartments that help degrade and recycle intracellular and extracellular metabolites with the help of acidic hydrolase enzymes (Settembre et al., 2013b). Several studies have suggested that any dysfunction in lysosomes may lead to neurodegenerative diseases such as AD, PD, and HD (Hussain et al., 2018; Koh et al., 2019; Malik et al., 2019). Therefore, the upregulation of lysosomal biogenesis, especially in microglia, may help in attenuating Aβ pathogenesis in AD. In this regard, TFEB may act as a potential target molecule as this is known to regulate lysosomal function by inducing genes related to lysosomal biogenesis and autophagy (Bala and Szabo, 2018). The beneficial effect of TFEB has been found capable of addressing Aβ pathology in AD mouse models (Polito et al., 2014). In APP/PS1 mouse model, expression of TFEB in astrocytes facilitated a reduction in Aβ plaques by enhancing their uptake and lysosomal degradation (Xiao et al., 2015). In addition, when TFEB was given intracranially by stereotaxic injection of adeno-associated virus (AAV)–TFEB in APP/PS1 mice, it reduces APP, Aβ production, and plaque deposition by enhancing autophagic flux via the endosome-lysosome pathway (Xiao et al., 2015).

Xiao et al. (2014) shows that TFEB enhances lysosomal biogenesis and contributes to an increased Aβ clearance and reduced Aβ generation in both astrocytes and neurons. In addition, TFEB was also discovered to protect against the toxicities of the aggregated proteins. Overexpression of TFEB in PD animal models led to ameliorating neuronal toxicity from α-synuclein and htt-aggregates (Decressac et al., 2013). In a study involving miRNAs in AD patients, miR128 was found to be upregulated in the hippocampus and monocytes (Lukiw, 2007). miR128 targeted TFEB, resulting in its downregulation and significant reduction of lysosomal enzymes and Aβ degradative capacity in AD patients. In monocytes of late-onset AD patients, this might have occurred due to targeting TFEB by miR128 upregulation, resulting in lower expression and decreases in TFEB transcripts and their nuclear localization. Similarly, in fibroblasts derived by patients with familial AD PS1 mutation, A246E displayed upregulation of autophagy–lysosomal pathway (ALP) genes, particularly TFEB (Coffey et al., 2014). Thus, exogenously induced expression of TFEB may act as an important target to ameliorate the disease. Despite these path-breaking observations, the underlying mechanisms behind TFEB working, especially its upstream regulation, remains to be elucidated.

## Effect of Transcription Factor EB on Tau Pathology in Alzheimer’s Disease

Tauopathies are a group of neurodegenerative diseases including AD and are defined by the insoluble filaments that accumulate as intracellular NFTs (Gao et al., 2018). These NFTs are composed of aggregates of abnormally hyperphosphorylated and misfolded tau protein. The major pathological hallmarks of related neurodegenerative diseases (Iqbal et al., 2010; Gao et al., 2018). Tau is usually localized in the neuronal axons, where it is involved in microtubule assembly and stabilization (Gendron and Petrucelli, 2009). At lower levels, tau helps in dendritic signaling. However, hyper phosphorylation causes dissociation of tau from microtubules resulting in its redistribution and forming aggregated deposits (Mietelska-Porowska et al., 2014). Tau is predominantly an intra-neuronal protein but is secreted and displaying the prion-like characteristics of Tau pathology (Brunello et al., 2020).

Today, there is no known effective treatment for any tauopathies. The available treatments aren’t effective in preventing or slowing the disease progression, as they don’t treat the root cause of these tauopathies.

The extracellular tau has been found to seed monomers to propagate the tau aggregates and is the prime target of the current immunotherapy (Xu et al., 2020). In this regard, several studies have found a link between TFEB and lysosomal exocytosis of tau proteins (Martini-Stoica et al., 2018). In a study, TFEB was found to regulate lysosomal exocytosis of tau and its loss of function in PS19 mice expressing mutant tau and exacerbated tau pathology associated with enhanced intra-neuronal pathology (Xu et al., 2020). Further, it was found that TFEB regulated the secretion of truncated mutant tau lacking microtubule binding region (MTBR) with the help of the lysosomal calcium channel, mucolipin transient receptor potential-1 (TRPML1).

TFEB activation also helped to reduce the accumulation of pathogenic protein aggregates in HD and PD models, suggesting its therapeutic role as target protein (Sardiello et al., 2009; Medina et al., 2011; Nnah et al., 2019). Since autophagy is the sole mechanism for the clearance and degradation of damaged organelles and misfolded proteins; and this is dysfunctional in AD and related tauopathies; we believe that TFEB, could ameliorate these pathological hallmarks of neurodegenerative diseases.

A study involving rTg4510 tau transgenic mouse found that TFEB effectively ameliorated phospho-Tau/NFT pathology, neurodegeneration, and behavioral deficits while exhibiting no adverse effects (Polito et al., 2014). TFEB was effective when introduced both before and after the onset of neuropathology. Besides the autophagy-lysosome pathway, substantial evidence also supports a non-cell-autonomous role of TFEB in cellular clearance through neuronal exocytosis and astroglia-mediated endocytosis. Therefore, it might be possible that TFEB promotes lysosomal exocytosis and subsequent astroglia uptake of tau and that TFEB-mediated glial uptake of extracellular tau prevent the cell-to-cell transfer of the NFT-like pathology. We need to look at this link as it may help us provide mechanistic and functional insights into astrocytes’ poorly defined role in tau clearance and the interplay between neurons and astrocytes in mediating tau pathogenesis.

## Prospects of Natural TFEB Modulators in Alzheimer’s Disease

Natural products may play an encouraging role in the treatment of AD and other neurodegenerative conditions. Currently, all drug-based measures are not true therapeutic options for AD (Di Paolo et al., 2019). Moreover, they are associated with several limitations such as toxicity, side effects, feasibility, expensive medication, and require prolonged administration. Since neurodegenerative diseases cause poor communications that might be cured with natural products, therefore, a natural therapeutic option that is readily available may play an essential role in controlling neurodegenerative disorders. Several studies have reported various natural products/compounds to possess different biological properties and have been found to interact and enhance the activity of TFEB. With the rapidly evolving identification and discovery of natural products showing promising activities against neurodegenerative diseases, neuroscientists have developed an interest in the neuroprotective potential of natural compounds like flavonoids, alkaloids, steroids, triterpenoids, quinones, terpenes, and lignans, etc.

**FIGURE 3:**
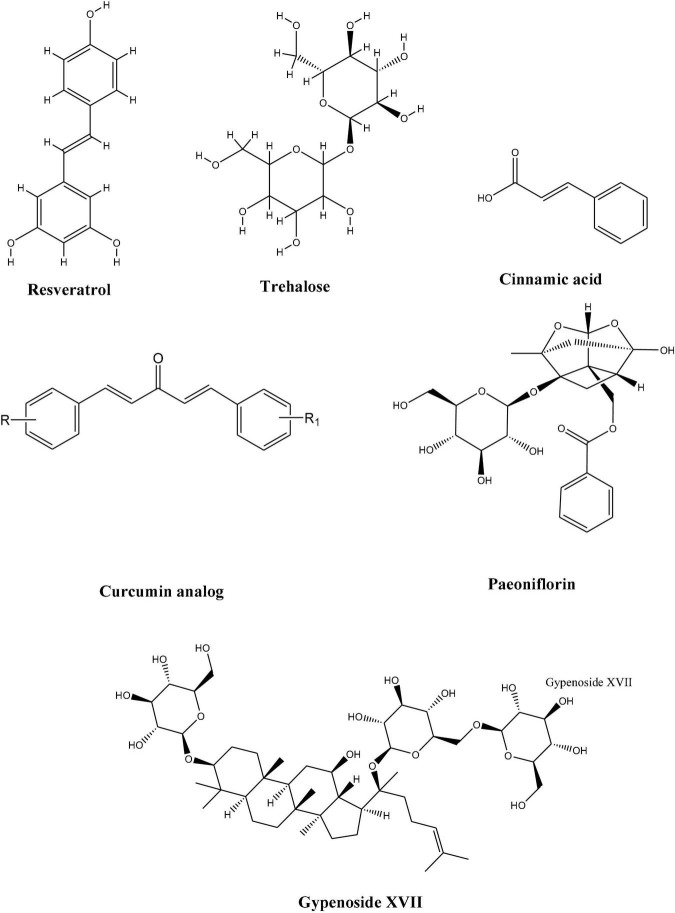
Chemical structures of natural compounds with therapeutic potential against neuro disorders.

Although many of these compounds have shown promising results, very few or none have reached the clinical level. This necessitates the adaptation of more collaborative efforts to bring and develop these compounds as a promising drug candidates. To address this, we will discuss different natural compounds obtained from natural sources which exhibited the potential to enhance TFEB activities and thus may have the therapeutic potential against neuro disorders (Figure 3).

### Paeoniflorin

Paeoniflorin (PF), a major component of *Paeonia* plants, was found to partly exert therapeutic effects on behavioral and pathological impairments in the SBMA mouse model. It upregulated molecular chaperons and TFEB expression, thus helping in protein clearance in motor neurons and muscles (Tohnai et al., 2014). Since progressive protein accumulation is a defining feature of many neurodegenerative disorders; the authors also found a simultaneous activation of molecules involved in the proteolytic machinery, i.e., molecular chaperone–ubiquitin-proteasome system (UPS) and the autophagy system by paeoniflorin. Paneoflorin upregulated the expression of nuclear factor-YA (NF-YA) which in turn upregulated the molecular chaperons and TFEB which thus helped in rescuing the behavioral and pathological impairments.

### Curcumin Analogs

**FIGURE 4:**
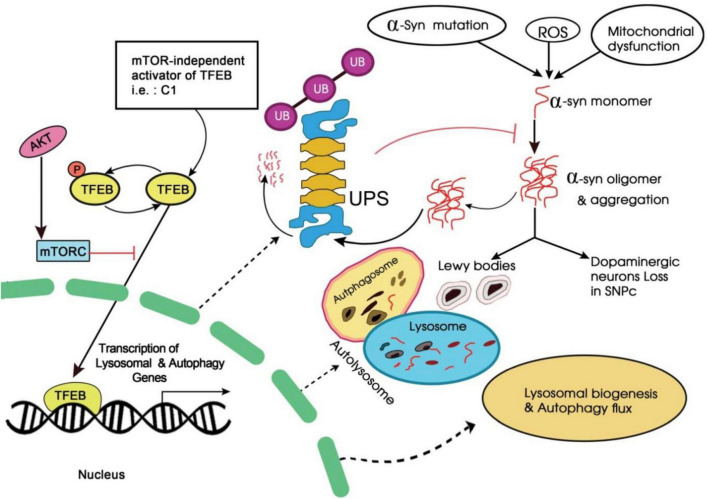
Schematic representation of phosphoinositide 3-kinase-AKT-MTOR signaling pathway.

Curcumin, a natural polyphenolic compound derived from Curcuma longa *Linn*. has been known to possess a wide range of pharmacologic activities (Amalraj et al., 2017). It is well known for its role in enhancing autophagy activity by inhibiting the phosphoinositide 3-kinase-AKT-MTOR pathway (Wang et al., 2019; Figure 4). In a study, a monocarbonyl analog of curcumin named C1 was identified as an mTOR-independent activator of TFEB. It specifically bound to TFEB and promotes its nuclear translocation (Song et al., 2016). Further, it was observed that by enhancing TFEB expression, C1 induced autophagy and lysosome biogenesis.

Since the compounds like C1 analog control TFEB activity independently of mTORC1 (mechanistic target of rapamycin (serine/threonine kinase) which is known to regulate multiple cellular processes; therefore these C1 analogs could play an important role in the treatment of neurodegenerative diseases (Martina et al., 2012).

### Cinnamic Acid

Cinnamic acid, a naturally occurring aromatic fatty acid found in several plant-based products. It is naturally synthesized in plants by enzymatic deamination of phenylalanine to form numerous phytochemical compounds such as coumarins, flavonoids, and lignins, etc. (Marchiosi et al., 2020). In multiple reports, cinnamic acid and its derivatives have been found to possess and display potent anti-microbial, anti-oxidant, anti-cancer, anti-atherogenic, anti-tuberculosis, and anti-fungal properties (Sova, 2012; Kumar and Goel, 2019). Keeping this, a study by Chandra et al. (2019) delineated that cinnamic acid acts as a ligand of peroxisome proliferator-activated receptor α (PPARα) and induces lysosomal biogenesis by activating PPARα to transcriptionally upregulate TFEB expression. Additionally, the administration of cinnamic acid in mice models of familial AD significantly reduced cerebral Aβ plaque accumulation and improved memory features. Therefore, the stimulation of lysosomal biogenesis by cinnamic acid may present therapeutic implications for treating all neurodegenerative disorders arising from the accumulation of toxic protein aggregates.

### Pomegranate Extract

The pomegranate (*Punica granatum*) is a small bush/tree in the family *Lythraceae*, subfamily *Punicoideae*; containing a high amount of polyphenols, condensed tannins, catechins, and prodelphinidins (Guerrero-Solano et al., 2020). In a study by Tan et al. (2019), pomegranate extract (PE) was found to activate TFEB via a novel regulatory pathway requiring cytosolic Ca^2+^ and was independent of ERK1/2-mTORC1 and calcineurin independent regulatory pathways. The activation of TFEB in turn resulted in upregulation of autophagy and lysosomal compartment in neuronal cells. PE also altered mitochondrial morphology and promoted autophagosome recruitment. Upon onset of mitochondrial stress, PE further augmented mitophagosome formation and engaged PTEN Induced Kinase 1 (PINK1) and Parkin to the mitochondria to induce mitophagy and negate ROS production and mitochondrial impairment (Tan et al., 2019). Recent studies have demonstrated that urolithin A; a metabolite of pomegranate polyphenol ellagitannins, enhances mitophagy (Cásedas et al., 2020).

### Phytoestrogens

In a study conducted by Meng et al. (2016), GP-17; a novel phytoestrogen isolated from ginseng, *P. notoginseng*, and *G. pentaphyllum* displayed neuroprotective efficacy against Aβ25–35-induced apoptosis in PC12 cells by modulating autophagy. Further, they identified the role of GP-17 in accelerating autophagic clearance through TFEB activation in mice models of AD (Meng et al., 2016). There was a significant acceleration in the autophagy-based support of Aβ-APP *in vivo* and *in vitro* through TFEB activation. GP-17 was found to help in the nuclear translocation of TFEB from TFEB/14-3-3 complexes which led to the induction of autophagy and lysosome biogenesis (Meng et al., 2016). The knockdown of TFEB finally validated this.

### Trehalose

Trehalose is a natural disaccharide that has been found to enhance the autophagic clearance of neurotoxic misfolded proteins which are prone to aggregate (Sarkar et al., 2007; Aguib et al., 2009). In experimental models of neurodegenerative diseases, trehalose has been found to ameliorate the disease phenotype of AD (Tanaka et al., 2004; Rodríguez-Navarro et al., 2010; Perucho et al., 2012). It stimulated the nuclear translocation of TFEB by indirectly controlling its phosphorylation via the adenosine 5’monophosphate-activated protein kinase pathway and AKT pathway and further regulated the expression of autophagy activation genes namely beclin-1 (BECN1), autophagy related 10 (ATG10), autophagy related 12 (ATG12), and SQSTM1/p62 as well as of PPARG coactivator 1 Alpha (PPARGC1A) which acts as both target and inducer of TFEB expression (Rusmini et al., 2019). Trehalose also modulated key components (HSPB8 and BAG3) of the CASA complex, which help deliver misfolded proteins to autophagosomes. However, a major drawback of trehalose is that it cannot be given orally because it is degraded by the hydrolyzing trehalose (TREH) enzyme present in the gastrointestinal tract. This suggests that trehalose could be potentially administered intravenously; however, the analogs of trehalose, melibiose, and lactulose which are resistant to intestinal TREH; act as potent mimics of trehalose and could be highly beneficial to the treatment of neurodegenerative disorders. Thus, trehalose might be a suitable alternative therapeutic option for neurodegenerative diseases because of its ability to regulate TFEB expression and thus could help restore normal lysosomal function.

### Resveratrol

Resveratrol is a stilbenoid that several plants produce in response to injury or under any attack by bacterial or fungal pathogens. It is naturally abundant in many plants such as grapes, blueberries, raspberries, mulberries, and peanuts (Abu-Amero et al., 2016). A study by Bao et al. (2016) acknowledged the use of Resveratrol in AD and found that it is a known activator of sirtuin 1 (SIRT1). In this study, the authors identified SIRT1 as a potential candidate for the deacetylation of TFEB at the K116 site. Further, when 293T cells were treated with resveratrol, it reduced TFEB acetylation. Subsequently, SIRT1 overexpression enhanced lysosomal function and fAβ degradation by upregulating transcriptional levels of TFEB downstream targets. The findings in this study reveal that Resveratrol; which activates SIRT1; which in turn deacetylates TFEB, could regulate lysosomal biogenesis and fAβ degradation making microglial activation of TFEB a possible strategy for attenuating amyloid plaque deposition in AD.

## Current Approaches to the Treatment of Parkinson’s Disease

Parkinson’s disease (PD) treatment has enormously progressed over the past half-century; still, levodopa remains the most potent drug to control PD symptoms (Jankovic and Aguilar, 2008). Before establishing medical therapy, an accurate diagnostic strategy for PD is of utmost importance (Massano and Bhatia, 2012). Each individual’s treatment is personalized, and various drugs, including levodopa, are currently accessible for the affected individuals (Jankovic and Aguilar, 2008). Dopamine agonists such as catechol-o-methyl-transferase (COMT) inhibitors and non-dopaminergic elements are the most common medications accessible for PD patients (Cooray et al., 2020). The top-to-bottom drug observations within each class are exceptional. PD is a widespread motor dysfunction disease and the second most extensive neurodegenerative disorder characterized by dopaminergic neuronal death (Rai et al., 2017, 2019; Birla et al., 2019; Singh et al., 2020a,b). Likewise, it is also identified by predominant neuronal loss in substantia nigra pars compacta (SNpc) and in the striatum of mid brain area (Prakash et al., 2014; Moors et al., 2017; Yadav et al., 2017), Various therapeutic targets are identified for the treatment of PD. Mucuna pruriens and some edible mushrooms offer significant Anti-Parkinsonian activity by acting on these targets (Rai et al., 2017, 2021).

The intracellular Lewy bodies’ inflation is enhanced in thread-like proteinaceous inclusions (called Lewy neuritis) and alpha-synuclein protein, known to induce the PD motor symptoms (Stefanis, 2012). However, genetic analysis characterizes the mutations in DJ-1 (PRAK7), alpha-synuclein, PINK1, ubiquitin-C-hydrolase, and community-based genetic mutations, i.e., Ashkenazi jews gene of glucocerebrosidase, that are directly linked with PD onset (Cooray et al., 2020). It is reported that mutations in signaling pathways i.e., Miro GTPase, Leucine-rich repeat kinase 2, show a crucial role in PD progression and their beginning (Singh et al., 2019). Also, L-DOPA, prevalently used medicine against PD, alternative physiological approaches with deep brain stimulations having network-centric therapy are used to prevent PD symptoms (such as trembling and rigidity) (Siddiqui et al., 2015). Moreover, Bourque and colleagues reviewed the hormone therapy and reported the potentiality of steroid hormones, particularly sex steroids, i.e., progesterone and estrogen therapy against PD (Bourque et al., 2019). The PD was also treated through the modulation of neurotransmitters of specific estrogen receptors (Siani et al., 2017). It is suggested that the optimized solutions should advance with presently available drugs to particular brain receptors target.

In addition to above mentioned therapeutic approaches a group of Japanese scientists performed the gene remodeling strategy (CRISPR) and induced pluripotent stem cells. The Ebben et al. (2011) later utilized this technique to demonstrate a potential therapeutic recovery in the Parkinson disease rat model. However, there is a need to intensify the clinical level investigations having a large cohort. The stem cell concepts were also successfully used on PD monkey models, but the ethical concern arises at hESCs at the most considerable extent of clinical trials. In addition, there are several challenges while building stem cell-based treatments, i.e., identification of suitable cell line, development of clinical administrable cell products, assurance of quality and control, and pre-clinical trials for proper dosage (Aly, 2020). More mediation is required to achieve a precise control level in dopaminergic neurodegeneration progression. Also, no authentic drug has been recognized to halt the neurodegenerative progression of PD (Moors et al., 2017).

### The Role of NF-κB in Parkinson’s Disease

The nuclear factor kappa-light-chain-enhancer of activated B cells (NF-κB) is dimeric transcription factor consist of five different proteins namely NF-κB1 (p50/p105), NF-κB2 (p52/p100), RelA (p65), RelB, and c-Rel proteins (Oeckinghaus and Ghosh, 2009). In RelA (p65), RelB, and c-Rel proteins, a trans-activated domain present in its C- terminus (Gilmore, 1999). Therefore, NF-κB1 and NF-κB2 proteins are synthesized like large precursors molecules such as p105 and p100, which go under the ubiquitination process to make mature NF-κB subtype, i.e., p52 and p50 (Beinke and Ley, 2004). The p105 and p100 proteins ubiquitination comprises discriminative degradation of C-terminus containing ankyrin repeats (Hoesel and Schmid, 2013). Several studies suggest that the gene expression of several pro-inflammatory reactions is maintained through the NF-kB transcription factor (Lawrence, 2009; Bhatt and Ghosh, 2014; Martins et al., 2016). The NF-κB is considered the master switch for many inflammatory mediators gene expression. The reaction of inflammatory factors, i.e., IL-1alpha, TNF-α, and IL-1beta having bacterial LPS and cell damage product, is arbitrated via NF-κB activation (Kany et al., 2019). Simultaneously, NF-κB shows a vital role in the inflammatory reaction via gene regulation that encodes chemokines like IL-1β, TNF-α, IL-12/23 and MCP-1 and cell adhesion molecules like E-selectin, ICAM-1, and VCAM], and NADPH oxidase subunit p47 and p77, and inducible nitric oxide synthase (Chen and Manning, 1995; Xia et al., 1997; Tak and Firestein, 2001; Gauss et al., 2007). The standard amino acids that are corticosteroids and salicylates utilized to treat inflammatory conditions exert anti-inflammatory responses via NF-kB inhibition (Auphan et al., 1995; Bantel et al., 2000). These compounds are considered potent inhibitors of microglial stimulation, and they have a neuroprotective impact on dopaminergic neurons approved in both *in vivo* and *in vitro* models. Hence, NF-kB responses appear as a unique target to control chronic inflammatory reactions, and NF-kB inhibition in microglia may lead to more efficient PD therapy.

### The Role of STAT3 in Parkinson’s Disease

The diverse pro-inflammatory and neurotoxic factors (IL-1-β, IL-6, NO, and TNF-α) are secreted through microglia activation (Hanisch, 2002; Smith et al., 2012). The pro-inflammatory cytokines such as TNF-α and IL-1β can stimulate microglia and respond to neuroinflammatory events (Bachiller et al., 2018). The cytokine’s early expression is induced through the mRNA stabilization or inflammatory mediators binding to Toll-like receptors. After releasing the cytokine, it stimulates the activation of signal transducer and activator of transcription 1/2 (STAT1 and STAT2) (Przanowski et al., 2014). Consequently, STAT3 is activated in induced microglia that drives the dopaminergic neurons against apoptosis (Tanaka et al., 2013). This process occurs via transcriptional stimulation of cell death arbitrating genes, i.e., caspases, BCL-XL, Fas, TNF-based apoptosis-induced ligand. The microglia STAT3 activation causes autophagocytic reactions of dopaminergic neurons leading to their attenuation; in an IL1-dependent fashion, thus resulting in PD (Tanaka et al., 2013). Therefore, STAT3 signaling cascade might also be employed for the treatment of PD.

### The Role of Transmembrane Receptors in Parkinson’s Disease

The type I Transmembrane receptors (TLRs) contain intercellular Toll/IL1 signaling domain and leucine-rich repeats. The molecules that are termed as pathogen-associated molecular patterns (PAMP) include lipoproteins, LPS, and flagellin with bacterial and viral genetic material are the significant legend for this receptor (Janssens and Beyaert, 2002). TLRs can initialize the signaling pathways that enhance the inflammatory mediator’s expression, cell adhesion molecules, and chemokines after molecular exposure of these molecules (Lai and Gallo, 2008). It is evidenced by health scientists that TLRs are highly expressed in activated microglia during dopaminergic neurons degeneration (Fiebich et al., 2018). The NF-kB canonical pathway is then induced via TLRs. Thus, activated NF-kB is then trans-located towards the nucleus, enhancing several pro-inflammatory molecular expressions (Lawrence, 2009). Pro-inflammatory elements released from stimulated microglia increase SNpc oxidative stress, resulting in neuronal degeneration of dopaminergic neurons (Peterson and Flood, 2012; Béraud et al., 2013). Therefore, synergistic targeting of TLRs and NF-kB might be a better option for PD therapeutics.

### The Role of AP-1 in Parkinson’s Disease

The activator protein-1 comprises Jun (i.e., Jun-b, Jun-D, and c-Jun) and Fos heterodimers (Fra1, Fra2, cFos, FosB) having stimulating transcription factors (Gazon et al., 2018). The AP-1 activity is induced through a mitogen-activated protein (MAPK) kinase cascade (Karin, 1995). Stimulation of MAPK cascade resulting in the activation of c-Jun N-terminal kinase, i.e., JNK, that phosphorylates c-Jun (Cargnello and Roux, 2011). Therefore, activated TLR in SNpc microglia can trigger the AP-1 activation. Thus activated AP-1 further induces the vicious neuroinflammation cycle in SNpc microglia that drives dopaminergic neurons to apoptosis (Tiwari and Pal, 2017). Nevertheless, the exact mechanisms in PD pathology are still not correctly elucidated. Thus, further studies are required to identify the convincing potential of AP-1 in PD therapeutics.

### The Role of FAF1 in Parkinson’s Disease

Fas-associated factor 1 (FAF-1) is is evolutionarily conserved as factor binding protein and product of the Parkinson’s disease 10 gene (PARK10) (Betarbet et al., 2008; Menges et al., 2009). This gene is located on chromosome 1p32 and associated with late-onset PD (Farrer et al., 2006). FAF-1 is categorized as the pro-apoptotic protein, which commutes cells across the apoptotic pathways. Evidence suggests that FAF-1 is overexpressed in the PD patients SNpc (Betarbet et al., 2008). The endogenous FAF-1 expression in SNpc can be stimulated via oxidative stress. Its overexpression also induces the dopaminergic neurons against apoptosis via promising caspase-3 activated cell death (Sul et al., 2013). Therefore, FAF-1 can also be targeted for the treatment of PD.

### The Role of p38 MAPK in Parkinson’s Disease

The MAPK makes special categories of serine/threonine kinases. The MAPK is divided into four distinct classes: JNKs, extracellular signal-related kinases, p38 MAPKs, and typical MAPKs i.e., ERK3, ERK5, and ERK8 (Cargnello and Roux, 2011). Due to extracellular stress and cytokines, the p38 MAPKs are activated and it is described as stress-induced protein kinases (Corrêa and Eales, 2012). The isoform of MAPK is p38α/β that binds explicitly and activates the MAPK APK-2 (MK2) and MAPKAPK-3 (MK3) serine/threonine kinases; it is the MAPK induced protein kinases, subfamily members (Roux and Blenis, 2004). Among all of the kinase groups activated by MAPK; isoforms (p38α/β) are the most significant due to their mediating role in inflammatory responses and cellular stress induction. External stimuli i.e., pro-inflammatory cytokines and stress, activate the p39 MAPK (Roux and Blenis, 2004). The complex of p38 MAPK-MK2 may promote the inflammatory responses; it is evident via MK2- knockout mice that resist the endo-toxic effect at the time of stimulation with LPS (Kotlyarov et al., 1999). MK2 also mediates the production of several inflammatory reactions (IL-6, IL-8, and TNF-α) with other cytokines showing a significant role in inflammatory responses. It is observed that in interferon-γ–stimulated microglial cells and LPS, the MK2 has increased expression that releases inflammatory mediators (Culbert et al., 2006). Several scientists reported that p38 MAPK-MK2 pathway activation subsequently enhances inflammatory cytokines thus might play an important role in PD (Bachstetter and Van Eldik, 2010).

### Therapeutic Role of Transcription Factor EB in Parkinson’s Disease

The TFEB is an essential helix-loop-helix zipper transcription factor and is currently a potential drug target to induce autophagy. It helps to control autophagy-driven gene expression in distinct autophagy stages along with autophagosome formation, fusion, and degradation (Zhao and Czaja, 2012; Cortes and La Spada, 2019). At the normal state, the TFEB is localized in the cytoplasm, and on receiving stress stimuli such as starvation, it translocates to the nucleus. The TFEB activation and its nuclear translocation may give strategy to autophagic stimulation (Chen et al., 2020). Evidence suggests that the mTORC1 regulates autophagy via TFEB nuclear location induction and in a mTORC1-dependent manner (Martina et al., 2012; Cortes and La Spada, 2019). The inhibition of mTOR is responsible for activating TFEB by promoting its nuclear translocation. Therefore, some low molecular weight compounds may directly target the TFEB for its increased autophagic reaction. Flubendazole has served in autophagosomal activation by the TFEB pathway that does not depend on TOR pathways (Brady et al., 2018). The TFEB attracts a lot of attention in its ability to stimulate the intercellular pathological factors in diverse murine models of AD and PD (Napolitano and Ballabio, 2016). It also suggests that the novel therapeutic strategy could depend on the initiation of TFEB action (Settembre et al., 2013a; Zhuang et al., 2020).

The TFEB can initiate autophagy and lysosomal biogenesis by binding with lysosome elements and coordinate its expression and regulation. Likewise, the proposed TFEB activations consider a strategy reversing PD and lysosome/autophagy dysfunction (Raben and Puertollano, 2016). Considerably, overexpression of TFEB has proven to have a neuroprotectiveness against the PD animal model which is based on the human α-synuclein over-expression (Torra et al., 2018). However, thousands of other genes that may control TFEB are associated with distinct biological pathways such as gene expression regulation, mitochondrial metabolism, cell cycle, translation, cellular stress response, and others. The TFEB overexpression and the activation of these distinct processes have never been identified for their neuroprotective impact. Indeed, the transcription factor of the microphthalmia family (MiT) that comprises TFEB a proliferative physiological regulator, differentiation, and various non-nervous tissue survival (Zhuang et al., 2020).

Moreover, TFEB member is studied in a very least amount. The TFEB overexpression affects the mouse substantia nigra dopaminergic neurons utilizing the adeno- linked viral vector such as AAV-TFEB (Torra et al., 2018). TFEB over expression might inhibit the dopaminergic neuronal loss or atrophy in toxin-induced PD model and can be effectively targeted for its treatment.

## Autophagy–Lysosome Pathway Activators From Natural Products to Treat Parkinson’s Disease

Since currently available PDPD medications are indicative and linked with several side effects, a natural therapeutic option that possesses significant neuroprotective and anti-aging properties could play an essential role in all forms of neurodegenerative disorders. In recent years natural products have emerged as potential alternative therapeutic agents for the treatment of neurodegenerative disorders such as PD (Angeloni and Vauzour, 2019; Birla et al., 2019; Sharifi-Rad et al., 2020). Targeting ALP has been currently proposed as an optimistic scheme for neurodegenerative diseases, including PD (Zhuang et al., 2020). However, the protective impacts of pharmacological and genetically induced ALP may have been studied in PD experimental model in recent years. Amidst these ALP small activators, molecules from neuro-protective herbal medicines are receiving enhanced attention. Several novel ALP activators have determined that they bring neuroprotective efforts in animal and cellular PD models. These herbal ALP activators may show leading products for novel drug development to treat PD. Following are examples of some common natural compounds for the treatment of PD.

### Uncaria Rhynchophylla

Uncaria rhynchophylla (Oxindole alkaloids) is a regularly used ancient Chinese medicine formulated to treat PD symptoms. Several neuro-autophagy mediators such as Oxindole alkaloids Cory B may induce autophagic reactions in neuronal cells in an mTOR-independent or dependent manner (Lu et al., 2012; Zheng et al., 2021). Further examinations have revealed that Cory B inhibits the interaction of SNCA-HMGB1 and enhances the HMGB1-Beclin-1 interaction to save the defective autophagic-mediated overexpression of α-synuclein (Song et al., 2014). It was also discovered that the Cory-B promotes clearance of A53T mutant and wild-type α -synuclein in Drosophila neuronal cells. Similarly, Cory an enantiomer of Cory B was found to induce autophagy in an mTOR-dependent manner and induces wild-type and mutant α-synuclein clearance (Chen et al., 2014). Likewise, the Tianma Gouteng Yin (TGY) is an ancient medicine extract that originated from China employ as a neuroprotective impact in cellular and animal PD models (Liu et al., 2015). Furthermore, autophagic magnifying compounds in the elixir can slightly be contributed to the distinguished protective effects.

### Conophylline

Conophylline is extracted from the tropical plant *Tavertaemontana divaricate* which can also be identified in the leaf extract of Ervatamia microphylla, a form of vinca alkaloid (Sasazawa et al., 2015). It is found to stimulate autophagy in neuronal and non -neuronal cell lines in an mTOR- dependent manner. Furthermore, via autophagic flux promotion; the aminophylline may promote α-synuclein aggregates degradation and decrease cell death rate which is induced through MPP neurotoxin. Therefore, conophylline might protect the death of dopaminergic neurons in the PD model.

### Curcumin and Derivatives

Curcumin is an active turmeric (Curcuma longa) compound and it is a polyphenol having several pharmacological impacts (Kocaadam and Şanlier, 2017). Researchers reported that curcumin has neuroprotective properties in PD experimental models with multiple mechanisms (Wang et al., 2020). It also prevents inflammatory responses and oxidative stress and inhibits α-synuclein fibrillation and aggregation (Wang et al., 2010). The autophagy-mediated activity of curcumin in cancerous cells is well explored, it also plays a role in PD treatment. It rescues for A53T α-synuclein-mediated toxic effects in *in vitro* assays (Sundaram et al., 2017). In AH-SY5Y cells; curcumin may overexpress A53T α-synuclein and also can restore autophagy via mTOR pathways inhibition to decrease the A53 α- synuclein accumulation. The experimentations suggest that curcumin can reduce the level of neurotoxicity via increasing autophagy-induced α-synuclein degradation. Low curcumin bioavailability and poor absorption may restrict its clinical implementation. Several scientists have analyzed various curcumin derivatives to enhance potency and bioavailability to examine potential ALP inducer and neuroprotective elements. The potent TFEB enhancer has been identified as C1 that directly binds with TFEB to activate without mTOR activity inhibition. However, the C1 compound stimulates the ALP. It deteriorates α-synuclein in the *in vivo* and *in vitro* models and can be utilized as a lead compound for further drug development of PD (Song et al., 2016). Parkinsonian neurotoxicity is also prevented by curcumin analog through the TFEB pathway (Wang et al., 2020).

### Resveratrol

The Resveratrol found in red grapes and wine is a polyphenolic stilbene (3,5,4′-trihydroxystilbene) (Perrone et al., 2017). The evidence suggests that Resveratrol has neuroprotective effects for PD, which differs in *in vivo* and *in- vitro* models (Saiko et al., 2008). However, the Resveratrol ameliorates both pathological variations and motor loss in MPTP-treated mice and it induces the autophagic deterioration of α-synuclein (Guo et al., 2016). Likewise, in the case of rotenone-based induction of the SH-SY5Y cell PD model, resveratrol has been found to prevent apoptosis via HO-1- based autophagy. It is also reported that Resveratrol can activate sirtuin 1 and AMPK to increase the level of autophagy (Lin et al., 2014). Therefore, symptomatic PD might be treated with the help of Resveratrol.

### Amurensin G

The amurensin G isolated from *Vitis amurensis* roots is a resveratrol tetramer (Huang et al., 2001). Currently, amurensin G could attenuate cell toxicity in the PD model via autophagy induction. Its treatment may inhibit the rotenone-based apoptosis and also decreases the level of α-synuclein in SH-SY5Y cells (Ryu et al., 2013). However, the Beclin-1 knockdown abrogates the amurensin G effect, indicating the amurensin G autophagy induction vi Beclin-1 dependent pathway.

### Trehalose

Trehalose is an α-linked disaccharide synthesized through several plants and fungi. It has been determined to increase aggregated protein’s clearance, such as mutant huntingtin and α- synuclein via stimulating mTOR-independent autophagy (Khalifeh et al., 2019). The neuroprotective impact of trehalose has been analyzed through *in vivo* and *in vitro* models of PD. However, balanced PC12 inducible cell lines overexpress the α-synuclein may treat with trehalose can significantly initiate the A30P and A53T α-synucleinmutant’s degradation (Lan et al., 2012). The trehalose is also considerably reported in the AVV1/2 A53T A53T α-synuclein PD rat model for its effects on α-synuclein clearance. Furthermore, a Lewy body mice model showed that the trehalose could enhance the level of SigmaR1 and HSP90 (chaperone molecules) (Khalifeh et al., 2019). It can also activate the TFEB that enhances the activity of ALP. Likewise, the trehalose induces the underlying autophagy mechanism is still unclear.

### Celastrol

Celastrol found in *Tripterygium wilfordii* extract is a form of triterpenoid. It has been reported to stimulate apoptosis and autophagy in the tumor cell through the ROS/JNK signaling pathways (Li et al., 2015; Cascão et al., 2017). At the same time, its neuroprotective impacts have been examined (Zhang et al., 2020). The celastrol can insulate SH-SY5Y cells against rotenone-based damage via increased autophagy (Deng et al., 2013). Thus, Celastrol might also be a potential target to prevent the progressive neurodegeneration of dopaminergic neurons in SNpc of PD.

### Kaempferol

Kaempferol is found in *Cuscuta Chinensis* and *Hypericum perforatum* in the form of flavonoid (Chen and Chen, 2013). Kaempferol, in cancer cells; stimulates autophagy via AKT and AMPK signaling pathways (Huang et al., 2013). It is reported that the kaempferol exerts neuroprotective impacts on the PD rotenone model through enhanced autophagy-based mitochondrial turnover (Filomeni et al., 2012). A limited study is available regarding the therapeutic potential of Kaempferol in the PD model. Further studies will be needed to analyze the therapeutic potential in PD.

## Conclusion

Transcription factors play a vital role in the pathogenesis of AD and PD. Various transcription factor-like NFκB, AP-1, Sp-1, PPARγ, Norf2, STAT3, FAF1, p38 MAPK can be effectively targeted to diagnose and treat AD and PD. Among different transcription factors, transcription EB (TFEB) is of utmost value and great importance. Recently, several researchers are interested to explore the role of this transcription factor in the effective treatment of AD and PD. TFEB involves in the pathogenesis of AD through amyloid pathways. Similarly, TFEB also takes part in the progression of PD via alpha-synuclein pathways. Targeting TFEB with the help of chemical compounds isolated from natural resources might significantly contribute to the treatment of AD and PD. Tacrine, Donepezil, Rivastigmine, Galantamine, and Memantine are important chemical compounds that exhibit vital responses in AD and PD. Drugs and synthetic chemicals belong to phenolics and polyphenolics class exhibits better therapeutic efficacy for the effective treatment of PD and AD. In addition, AMPK-mTOR-TFEB axis also mediated the lysosmal dysfunction in neurodegenerative diseases as suggested by a number of researchers wordwide. miRNA are also involved in the TFEB mediated regulation of AD and PD. Furthermore, gut microbiome and are also connected with TFEB mediated autophagy in PD and AD. Further research will be needed to explore the role of TFEB in AD and PD.

## Author Contributions

SR, NT, PS, and DM: helped in the preparation of manuscript. AS and EH: helped in preparation of figures and tables. EV and MS: edited the whole manuscript. All authors contributed to the article and approved the submitted version.

## Conflict of Interest

The authors declare that the research was conducted in the absence of any commercial or financial relationships that could be construed as a potential conflict of interest.

## Publisher’s Note

All claims expressed in this article are solely those of the authors and do not necessarily represent those of their affiliated organizations, or those of the publisher, the editors and the reviewers. Any product that may be evaluated in this article, or claim that may be made by its manufacturer, is not guaranteed or endorsed by the publisher.
